# Reporting Guideline for Therapeutic Immersive Virtual Reality Interventions: International eDelphi Study (INVIRTUE)

**DOI:** 10.1177/29941520251404744

**Published:** 2025-12-29

**Authors:** Syl Slatman, Daniel Harvie, Max van der Heijden, Loes Bulle, Beate Dejaco, Harry van Goor, Tjitske Diederike Groenveld, Peter Alexander van de Hoef, Maaike Kragting, Raymond Ostelo, Brennan Spiegel, J. Bart Staal, Zina Trost, Darcy Ummels, Jesper Knoop, INVIRTUE Expert Panel

**Affiliations:** 1 Musculoskeletal Rehabilitation Research Group, School for Allied Health, HAN University of Applied Sciences, Nijmegen, The Netherlands.; 2 Research Group Innovation of Human Movement Care, Knowledge Center Healthy and Sustainable Living, HU University of Applied Sciences, Utrecht, The Netherlands.; 3 Innovation, Implementation and Clinical Translation in Health (IIMPACT in Health), Allied Health and Human Performance, University of South Australia, Adelaide, Australia.; 4 Research Group IT Innovations in Health Care, Windesheim University of Applied Sciences, Zwolle, The Netherlands.; 5 Department of Surgery, Radboud University Medical Center, Nijmegen, The Netherlands.; 6 Department of Physical Therapy, Research Centre for Health Care Innovations, Rotterdam University of Applied Sciences, Rotterdam, The Netherlands.; 7 Department of Health Sciences, Faculty of Science and Amsterdam Movement Science Research Institute, Vrije Universiteit, Amsterdam, The Netherlands.; 8 Department of Epidemiology and Data Science, Amsterdam UMC location Vrije Universiteit & Amsterdam Movement Sciences, Musculoskeletal Health, Amsterdam, The Netherlands.; 9 Department of Medicine, Cedars-Sinai Center for Outcomes Research and Education, Cedars-Sinai Medical Center, Los Angeles, California, USA.; 10 Karsh Division of Gastroenterology and Hepatology, Department of Medicine, Cedars-Sinai Medical Center, Los Angeles, California, USA.; 11 Radboud Institute for Health Sciences, IQ health, Radboud University Medical Center, Nijmegen, The Netherlands.; 12 Department of Psychological and Brain Sciences, Texas A&M University, College Station, Texas, USA.; 13 Department of Rehabilitation Medicine, Care and Public Health Research Institute, Faculty of Health, Medicine & Life Sciences, Maastricht University, Maastricht, The Netherlands.; 14 Amsterdam Movement Sciences, Program Musculoskeletal Health, Faculty of Behavioral and Movement Sciences, Vrij Universiteit, Amsterdam, The Netherlands.

**Keywords:** virtual reality, intervention reporting guideline, Delphi study, health care

## Abstract

With the rising adoption of virtual reality (VR) in health care, research publications on the topic are increasing rapidly. Guidance on reporting VR interventions is limited, which risks misinterpretation of findings, inappropriate data synthesis in reviews and meta-analyses, and reduced reproducibility. This could also result in possible misuse of VR interventions and might hinder further implementation in clinical practice. The aim of this study was to develop a reporting guideline for therapeutic, immersive VR interventions using head-mounted displays in scientific publications. To reach this aim, we intended to achieve expert consensus on key items and categories for reporting these interventions. We conducted a three-round, modified, online Delphi study with a validation meeting to develop a VR intervention reporting guideline. Participants were international experts in therapeutic VR research, working as academic or industrial researchers. Convenience, purposive, and snowball sampling were used to recruit participants. Consensus was defined *a priori* as ≥75% agreement on each reporting item and category. Of the 280 invited experts, 61 participants (22%) completed all three rounds of this Delphi study. Participants (32 females [52%]) had an average of 6 years’ experience with VR research and diverse backgrounds in VR research settings and disciplines. The final consensus-based VR intervention reporting guideline consists of 16 reporting items divided into six categories (i.e., theory, content, deployment, development, safety, and context). The INVIRTUE intervention reporting guideline provides a framework for describing therapeutic VR interventions. Using this tool may enhance the uniformity and completeness of VR intervention reporting and support study replicability and scientific progress. The reporting guideline will also facilitate efficient empirical comparison across existing VR interventions and ultimately contribute to the appropriate use and effective implementation of therapeutic VR in clinical practice.

## Introduction

Extended reality (XR) is increasingly used in health care as an (adjunct) intervention.^1^ XR is an umbrella term that encompasses virtual reality (VR), mixed reality, and augmented reality. VR can be defined as a “*computer-generated digital environment that can be experienced and interacted with as if that environment was real*”^2^ and is usually provided through a head-mounted display (HMD). By using an HMD, the user generally is sensory immersed in the virtual environment.^3^ Therapeutic VR is used for a variety of conditions, including (chronic) pain,^4^ phobias,^5^ and stress.^6^

Recently, a cross-sectional study reviewed the quality of reporting XR interventions in health care publications and showed that current intervention reporting is very poor.^7^ This corresponds with a recent review on XR interventions in pain management research, which showed that only 23% of the examined scientific papers included a substantial content description of the VR intervention, and only 46% included a substantial hardware description.^8^ This lack of detail and completeness in VR intervention reporting can lead to, for example, inappropriate interpretation, as well as comparisons and pooling of heterogeneous interventions in reviews and meta-analyses.^9,10^ This is a potential factor that hinders the adoption of therapeutic VR in health care settings and the replication of VR effectiveness studies.^11^ Recent literature on the current state of clinical VR research described it as “*the Wild West*,” with a lack of clear guidelines, standards, or substantial progress.^12,13^ Multiple sources suggest that the current state of VR scientific inquiry is underpinned by the absence of consensus on an intervention reporting guideline. This was mentioned as a limitation of recent VR research in different health care settings, including psychology,^14^ chronic pain,^15^ and physical rehabilitation research.^16^

Several intervention reporting guidelines of therapeutic interventions exist; the Template for Intervention Description and Replication (TIDieR) checklist is one of the most commonly used in health care.^17^ This checklist includes items delineating why, how, and where the intervention was delivered. More specific intervention reporting guidelines were developed for exercise interventions such as the Consensus on Exercise Reporting Template,^18^ but not yet for therapeutic VR. Given the multiple specific technical, content, and user-interactive elements involved in VR applications, existing therapeutic reporting guidelines are limited in their ability to capture the essential components of therapeutic VR interventions.^7^

The aim of this study was to develop an internationally accepted reporting guideline on how to describe therapeutic VR interventions in scientific papers. In this study, therapeutic VR is considered to be sensory immersive and provided through an HMD. We chose to focus specifically on VR rather than other forms of XR because it is the most extensively researched area within the XR domain.^8,19^ To reach this objective, we aimed to achieve consensus on key items and categories for reporting therapeutic VR interventions.

## Methods

### Design

The three-round, modified, electronic Delphi (eDelphi) survey and additional validation meeting was conducted among international VR research experts. Delphi techniques are often used to identify consensus or develop standards by collecting knowledge from domain-specific experts.^20,21^ Traditional Delphi studies use open-ended questions to identify responses for future Delphi rounds, while modified Delphi studies provide a set of initial statements that are developed *a priori*.^22^ Consequently, in modified Delphi studies, the coordinating researchers have an active role in generating consensus and generally two to three Delphi rounds suffice, rather than at least four rounds required in traditional Delphi studies.^21^ We chose a modified Delphi design to facilitate timely study completion, diminish burden on study participants, and permit tailoring of the process to study needs. In addition, existing intervention reporting guidelines served as a useful foundation for determining key items and categories. This Delphi study included three rounds: round 1 (demographic information, rating, and proposing [new] items and categories), round 2 (rating and testing stability of items and categories), and round 3 (rating and testing stability of items and categories). The reporting guideline was finalized during a validation meeting.

The study was based on the Enhancing the QUAlity and Transparency Of health Research (EQUATOR) network recommendations for developing reporting guidelines^23^ and reported according to the guidance on Conducting and REporting DElphi Studies guidelines.^24^

Approval to conduct the study was granted by the ethics committee of the HAN University of Applied Sciences (HAN ECO: 564.06/24). All participants provided electronic informed consent before participating in the study. The study was registered prospectively on the Open Science Framework (OSF) (doi: 10.17605/osf.io/hfp4m) and with the EQUATOR network. The Delphi rounds were conducted between June 2024 and March 2025, and the validation meeting was conducted in June 2025.

### Contributors

The study was primarily conducted by a day-to-day committee, supported by an international steering committee. Members of both committees were all researchers studying the application of therapeutic VR in health care interventions.

#### Day-to-day committee

The day-to-day committee of the Delphi study consisted of S.S., M.v.d.H., H.v.G., R.O., J.B.S., and J.K. The committee conceptualized, designed, coordinated, and coauthored this scientific paper on the Delphi study.

#### Steering committee

The international steering committee of this Delphi study consisted of D.H., L.B., B.D., T.D.G., P.A.v.d.H., M.K., J.B.S., Z.T., and D.U. The committee supported the day-to-day committee in providing feedback and coauthored this scientific paper.

#### INVIRTUE expert panel

Potential candidates for the expert panel were identified by the day-to-day committee. Expert panel members studied VR interventions in different health care areas and were included if they (1) (co)authored a scientific paper on therapeutic VR in a peer-reviewed journal and (2) were employed as researchers in an academic institution or industry. Participants were recruited through convenience sampling (e.g., by inviting colleagues of committee members), purposive sampling (e.g., by searching scientific databases for specific researchers), and snowball sampling (e.g., by asking participants to propose other participants in the first Delphi round). We strived to include at least 40 experts in the final round of the Delphi study, which is considered to be sufficient.^25^ Participants in the expert panel who completed all three Delphi rounds were mentioned as final authors of this article as part of the “VR intervention reporting international consensus (INVIRTUE) group” (see section “Acknowledgments”).

### Procedure and analysis

#### Preparation

Before the first round, a list of reporting items and categories was developed. The list was based on thematic analysis of >100 scientific papers that reported on the effectiveness of a therapeutic VR intervention (derived from Trost et al. [2021]^26^), other intervention reporting guidelines (i.e., TIDieR^17^ and RATE-XR^27^ checklist), and group discussions with the day-to-day and steering committees. Relevant features describing the therapeutic VR intervention of the aforementioned sources were systematically reviewed and inductively coded by one researcher (M.v.d.H.) and subsequently discussed with two additional researchers (S.S. and J.K.). Next, these three researchers categorized the codes into themes through iterative comparison and discussion.^28^ The *a priori* constructed list consisted of 26 intervention reporting items divided into five categories (i.e., theory, dose, content, preparation, and additional).

Before conducting the Delphi rounds, pilot tests were conducted to ensure study procedure feasibility. Results of the pilot tests were included in the final analysis, as no major issues were identified. Participation in the study was confidential, but not anonymous, because the coordinating researcher could identify participants based on their email address. Participant identification was necessary to be able to invite participants who completed a Delphi round for the next round. Personal data were stored on a secure server.

#### Round 1

All potential candidates of the expert panel were invited by the day-to-day committee through email to participate in round 1 of the study. The email contained general information about the Delphi study and a link to the survey on the web platform Qualtrics (Qualtrics, Provo, USA).

This first round included three parts. The first part contained the information letter and accompanied informed consent (Appendix A). After agreeing to participate in the study, participants continued to the actual survey. The second part requested the following (demographic) information about the participants: email address, age, sex, country of residence, VR research context, primary VR research area, and number of years’ experience in VR research. The third part comprised the *a priori* constructed list of 26 intervention reporting items with explanation per item and five categories (Appendix B), which were rated on a 9-point Likert scale per item (1 = of no importance to 9 = extremely important). The rating was based on the following question: “How important do you think this topic is for a therapeutic VR intervention reporting guideline?” Besides rating, there were two types of free-text boxes, one to comment on individual reporting items and suggest new items, and one to comment on the categories suggested in the *a priori* constructed list.

Participants received an email reminder to participate in the study after 7 and 14 days. Three weeks after the study started, this first round was closed. Items were proposed in the next round for inclusion in the reporting guideline when 75% of the participants rated an item 7 or higher, and less than 15% of the participants rated the item 3 or lower. Items were proposed in the next round to be excluded from the reporting guideline when 75% of the participants rated an item 3 or lower, and less than 15% of the participants rated the item 7 or higher.^29^ Item and category clarifications or modifications and new items proposed by participants were screened and analyzed inductively by the day-to-day committee and, if deemed of added value, included in the second round. This ensured that expert input, not just quantitative consensus, played a central role in shaping the subsequent rounds.

#### Round 2

The second round provided controlled feedback in the form of an anonymized summary of results from round 1. This round had three parts. First, to study item stability, items from round 1 that scored as sufficient to be included or excluded were presented with the question of whether the intervention reporting item should indeed be included or excluded in the reporting guideline.^21^ An item was deemed stable if 85% of the participants agreed that an item should be included or excluded.^30^ Second, items and categories without consensus were presented again with the median and interquartile range scores that the item scored in round 1.^31^ Third, new items proposed during round 1 were presented. Both items without consensus and new items were again rated based on the procedure presented for round 1. There was no option to suggest new items in this or subsequent rounds, but participants had the option to suggest minor revisions for both the items and their descriptions.

#### Round 3

The procedure of round 3 was similar to the survey procedure of round 2 and was based on the results from round 2. In addition, participants were asked to classify all proposed intervention reporting items in the following categories: “essential in reporting guideline,” “nice to have in reporting guideline,” or “not necessary in reporting guideline,” based on Duncan et al. (2020).^32^ Intervention reporting items were considered as included in the final reporting guideline if they scored a median rating of 7–9^33^ and/or if >50% of participants categorized an item in the “essential in reporting guideline” category. Similarly, intervention reporting items were considered excluded from the final reporting guideline if they scored a median rating of 1–3 and/or if >50% categorized an item in the “not necessary in reporting guideline” category.

#### Validation meeting

Results of round 3 were discussed with members of the day-to-day and steering committees during an online consensus meeting. In this meeting, each individual reporting item and associated category was discussed, and the members were asked to improve, as necessary, the information regarding the suggested reporting items (i.e., reporting item title, category, description, and example). The results of the consensus meeting formed the validated version of the VR intervention reporting guideline.

## Results

Of the 280 international VR research experts were invited for the first Delphi round, 61 (22%) completed all three Delphi rounds (see Table 1).

**Table 1.** Flow of Participants

	First round	Second round	Third round
Invited	280	104	77
Responded as unavailable	29	4	4
Started survey	128	86	65
Completed survey	104	77	61

The 61 participants who completed the third round had a mean age (standard deviation) of 39 (9.7) and an average of 6 years’ experience with VR research. They were located in 13 countries from 4 continents and studied VR in a variety of disciplines and settings. For more information on participant demographics information, see Table 2. Comparison between the participants who completed all three rounds and non-completers showed no large differences in demographic information (see Appendix C).

**Table 2.** Participant Characteristics

	Expert panel (*n* = 61)
Sex, female, *n* (%)	32 (52)
Age, mean (SD)	38.6 (9.7)
Country of origin, *n* (%)	
The Netherlands	26 (43)
The United Kingdom	7 (11)
The United States	5 (8)
Germany	4 (7)
Belgium	4 (7)
Australia	3 (5)
Switzerland	3 (5)
Denmark	2 (3)
Ireland	2 (3)
Italy	2 (3)
Canada	1 (2)
Chile	1 (2)
Poland	1 (2)
VR research primary area, *n* (%)^a^	
Pain management	24 (39)
Physical rehabilitation	20 (33)
Mental health therapy	15 (25)
Other^b^	24 (39)
VR research setting, *n* (%)^a^	
Out-hospital	42 (69)
In-hospital	32 (52)
Fundamental	19 (31)
Years’ VR experience, mean (SD)	6.0 (4.3)

a Multiple options possible.

b For example, cognitive rehabilitation.

SD, standard deviation; VR, virtual reality.

**FIG. 1.**
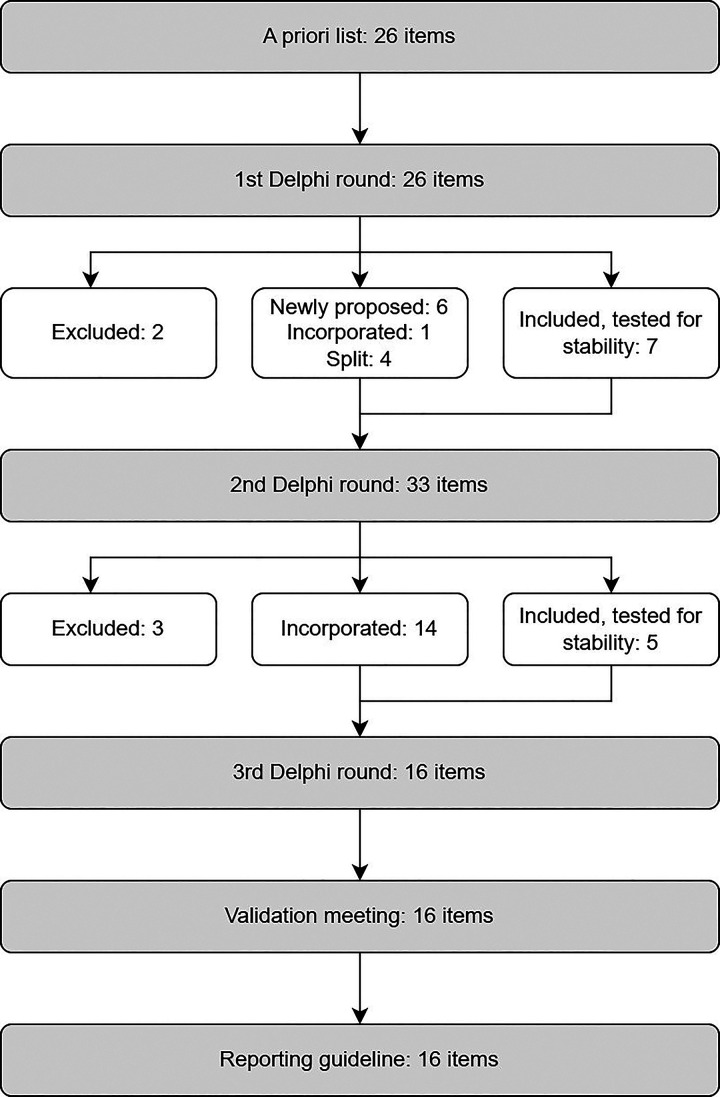
Flowchart of reporting items.

The flow of the included, excluded, new, incorporated, and split reporting guideline items throughout the three Delphi rounds and validation meeting is shown in Figure 1.

In the first round, the 26 items (divided into five categories) from the *a priori* constructed list of intervention reporting items were rated. After this round, seven items (i.e., *clinical aim*, *time per session*, *intervention duration*, *frequency*, *intervention setup*, *application/game*, and *interactivity*) were rated sufficiently important to include in the reporting guideline, six new items were proposed (i.e., *purpose of VR intervention*, *scientific evidence*, *participant involvement*, *availability/accessibility*, *professional training*, and *data security*), and two items were excluded (i.e., *intensity* and *software for development*). *Intervention procedure* was incorporated into the existing reporting item *application/game*, and four items were split (i.e., *proposed working mechanism* was split into *clinical aim* and *proposed working mechanism*; *hardware* was split into *technical requirements* and *hardware*; *intervention instruction* was split into *preparation time* and *intervention instruction*; and *intervention time session* was split into *time per session* and *VR time*).

In the second round, 33 items (divided into six categories) were rated based on the results of the first round. Three new categories (i.e., usability, population, and safety) were added, and two categories (i.e., preparation and additional) were removed. The 33 items of the second round included the 7 items that were included after the first round to test their stability. After this round, five items were rated sufficiently important to include in the reporting guideline (i.e., *clinical aim*, *application/game*, *interactivity and feedback*, *dosage*, and *setting and supervision*), and three items were excluded (i.e., *costs*, *presence*, and *embodiment*). Fourteen items were incorporated into existing reporting items (i.e., *purpose of VR intervention* was incorporated into *clinical aim*; *scientific evidence* was incorporated into *proposed working mechanism*; *feedback mechanism* was incorporated into *interactivity and feedback*; *intervention modification* was incorporated into *modification*, *personalization*, and *tailoring*; *motion tracking*, *audio*, and *technical requirements* were incorporated into *hardware*; *time per session*, *intervention duration*, and *frequency* were incorporated into *dosage*; *intervention setup*, *body position*, and *safety measures* were incorporated into *setting and supervision*; and *provision/delivery* was incorporated into *instruction*).

In the third round, 16 items (divided into six categories) were rated based on the results of the prior rounds and included in the reporting guideline. One category was added (i.e., deployment), and one category was removed (i.e., dose).

In the validation meeting, these 16 items were discussed, and their descriptions were improved if necessary (e.g., including more extensive examples per reporting item).

The final consensus INVIRTUE reporting guideline on how to describe therapeutic VR interventions contains 16 items divided among six categories: theory, content, deployment, development, safety, and context. The full version of the reporting guideline is shown in Table 3, and Appendix D provides a customizable version of the guideline. Appendix B gives a full overview of the reporting items and categories per round.

**Table 3.** Final INVIRTUE Reporting Guideline

	Item	Description	Example
Theory	1. Clinical aim	Description of the clinical aim of the VR intervention	The aim of the VR intervention was to increase the range of motion of the user’s shoulder
	2. Proposed working mechanism	Description of the proposed underpinning neuro/psycho/biological working mechanism of the VR intervention	The proposed working mechanism of the VR intervention was distraction
	3. Proposed user group	Description of whether the VR intervention was developed for a particular user group or body region	The provided VR intervention was originally developed for users with chronic low back pain
Content	4. Application/game	Description, illustration, and name of the VR intervention	The VR intervention “Beatsaber” was used (see Fig. 1). In this intervention, users use VR controllers to slice through flying colorful blocks in sync with music
	5. Interactivity and feedback	Description of whether and how the VR intervention was interactive and provided (audio-visual, tactile, or vestibular) task feedback	The VR intervention included interaction with avatars and provided audio-visual feedback
	6. Tailoring	Description of whether and how the VR intervention had the option to be tailored (e.g., using different levels or personalization) to the specific user	The VR intervention tailoring was possible through automatic progression in difficulty based on the performance of the user
	7. Availability	Description of whether the VR intervention is available to the public (open source or not)	The VR intervention used was an available off-the-shelf intervention
Deployment	8. Hardware	Description of the hardware and services (e.g., Wi-Fi) used, including HMD, audio, and motion tracking equipment	The VR intervention was provided using the standalone Oculus Quest 3 HMD VR headset with built-in spatial audio
	9. Dosage	Description of the VR intervention duration, frequency, and time per session	The VR intervention had a duration of 4 weeks, three times per week for 15 min per session
	10. Setting and supervision	Description of the location where the VR intervention took place and whether this was supervised	The VR intervention was provided in a hospital room supervised by a physiotherapist
	11. Instruction	Description of what kind of instruction was given to the user about the use of the VR intervention, by whom, and the time needed for the instruction	Users who received the VR intervention were given a 10-min verbal instruction by a nurse
	12. Preparation time	Description of how much time the setup of the system took before the VR intervention could start	Setup of the VR intervention took approximately 15 min
	13. Professional training	Description of the training given to the professional providing the VR intervention	Physiotherapists who provided the VR intervention received a 1-h online training
Development	14. End-user involvement	Description of whether the end-user (e.g., user or provider) was involved in the development of the VR intervention and in which way they contributed	Users with agoraphobia contributed to the intervention content description
Safety	15. Anticipated adverse events	Description of issues that may be encountered during the VR intervention and the utilized safety considerations	To diminish the risk of falling during the VR intervention, a safety harness was used
Context	16. Co-interventions	Description of other interventions provided during the VR intervention period	The users who received the VR intervention simultaneously received weekly psychotherapy

HMD, head-mounted display.

## Discussion

This Delphi study reached agreement on reporting items and associated categories that resulted in the consensus INVIRTUE intervention reporting guideline, which could improve uniformity and completeness of therapeutic VR intervention reporting in scientific papers. The consensus reporting guideline consists of 16 items divided into six categories.

### Discussion of intervention reporting guideline content

The Delphi rounds facilitated important discussions and revealed challenges in defining the final reporting guideline. One unexpected challenge was orienting all participants to the discussion of intervention reporting rather than traditional article reporting. This is reflected, for example, in participants suggesting including an item on adverse events, even though this would be more suitable for article results instead of the intervention description. The final reporting guideline includes an item on anticipated adverse events and safety considerations to prevent these (item 15). Another topic where consensus proved difficult was how detailed the reporting guideline should be. Some participants suggested including detailed technical reporting (e.g., utilized HMD refresh rate). This level of technical detail, while essential for hardware and software design, was generally considered less relevant for reporting of applied therapeutic VR research. After the three rounds, this item was excluded from the reporting guideline, but we urge authors to include this if they think it is appropriate. Further, the *a priori* list of reporting items included 26 reporting items, and after the first Delphi round, only six new items were proposed. This suggests that the initial list was already quite comprehensive and captured substantial relevant content. There was likewise some debate on using the term *tailoring* or *personalization* to describe reporting for item 6. In line with the TIDieR checklist and a recent review on the concepts of personalization and tailoring in eHealth interventions, we chose the term *tailoring* as we think this best reflects the description of the reporting item.^34^ Finally, although participants struggled to reach consensus on certain reporting items in the first and second rounds, consensus was reached on all items after the third Delphi round. This could be due to the novelty of the field and the broad range of therapeutic VR applications, which is reflected in other current debates in VR research, including the struggle to define (immersive) VR in general.^35–37^

### Comparison with other reporting guidelines

This is the first guideline specifically for VR intervention reporting, whereas more general article and intervention reporting guidelines already exist. Comparing our reporting guideline to the established TIDieR reporting checklist for interventions in general, it seems our reporting guideline is comprehensive.^17^ Most items of the TIDieR checklist are reflected in our reporting guideline, that is *brief name* (item 4 of our reporting guideline), *why* (items 1 and 2), *what* (items 4–8 and 11), *who provided* (items 10 and 13), *how* (item 10), *where* (item 10), *when and how much* (item 9), *tailoring* (item 6), and *modifications* (item 6). The only item not reflected in our reporting guideline is the final item of the TIDieR checklist (i.e., *how well*), which describes the planned and actual intervention adherence and fidelity. This is possibly due to the fact that this item is not strictly about describing the intervention itself but rather about the correct deployment of the intervention. Our reporting guideline is specifically designed for therapeutic VR interventions, providing more specific guidance. This is, for example, illustrated in the *why* reporting item of the TIDieR checklist (i.e., describe any rationale, theory, or goal of the elements essential to the intervention). Our reporting guideline divides this item into multiple items that ask for a therapeutic VR-specific clinical aim (item 1) and proposed working mechanism (item 2). Similarly, the intervention items of the RATE-XR checklist^27^ are reflected in our reporting guideline, that is, *4—setting and location* (item 10), *5b—providers and training* (items 10 and 13), *6—application description* (items 4–12), *7—intervention development* (item 14), and *8—participant timeline* (item 16). The RATE-XR article reporting guideline aims to improve the reporting of XR evaluation studies and includes items from the title to statements. However, the RATE-XR focus was not specifically on the in-depth description of therapeutic VR interventions.

### Intervention reporting guideline use

Sole use of this reporting guideline may not suffice for uniform and complete VR intervention reporting and possible study replication. It should be noted that this reporting guideline is not exhaustive, and additional details are warranted to ensure an in-depth and comprehensive intervention description for individual VR interventions. However, not all suggested reporting items are always necessary to report in a scientific paper, for example, when a VR intervention is described in-depth in a separate paper. In such cases, it is crucial that authors explain why they omitted this reportable information or where more information can be found. Unfortunately, it is not possible to establish a single, definitive reporting guideline that fits all VR interventions, given the considerable breadth of the therapeutic VR research field, which spans among others, physical, psychological, and cognitive rehabilitation applications. Moreover, it should be stressed that this reporting guideline focuses specifically on VR intervention description in a scientific paper. Other guidelines were developed to provide general guidance across a variety of scientific reporting, for example, the Consolidated Standards of Reporting Trials reporting guideline for randomized trials^38^ and Preferred Reporting Items for Systematic Reviews and Meta-Analyses reporting guideline for systematic reviews and meta-analyses.^39^ Also, the RATE-XR article reporting guideline was recently published to report early-phase clinical evaluation of XR applications.^27,40^ Therefore, we suggest combining the INVIRTUE intervention reporting guideline with a fitting article reporting guideline.

### Strengths and limitations

This study has several strengths. First, the study employed a comprehensive methodological approach including a literature review, multiple Delphi rounds, and a validation meeting, resulting in rich qualitative and quantitative data. Also, the international EQUATOR network recommendations to develop intervention reporting guidelines were adopted, which aim to advance high-quality reporting of health research studies.^41^ Furthermore, the study was registered *a priori* on OSF and the EQUATOR network. Second, the included expert sample was larger than anticipated and sufficient for a Delphi study.^25^ Also, no considerable differences in characteristics were observed between responders and nonresponders, suggesting decent external validity. The sample further included experienced VR experts with a large heterogeneity in locations, expertise, and backgrounds, which ensured comprehensive and trustworthy study results.

This study has the following limitations. First, participants were mostly from Western countries and especially from the Netherlands, since most members of the day-to-day and steering committee were from the Netherlands. In addition, the study lacked input from lower- and middle-income countries even though experts from these countries were invited. Consequently, the guideline may not reflect a global perspective on a VR intervention reporting guideline and, for example, may miss important topics like the availability of therapeutic VR interventions in different languages. Moreover, differences across countries in, for example, health care infrastructure and access to VR may limit the generalizability and global applicability of the reporting guideline. Second, since participants were invited through convenience, purposive, and snowball sampling, selection bias might have influenced the results. Also, using the *a priori* constructed list of reporting items may have introduced confirmation bias, which we mitigated by incorporating open-ended feedback and revising items across rounds. Third, we limited participants to those who (co)authored a scientific paper on therapeutic VR in a peer-reviewed journal, which may have overlooked variability in research expertise. Accordingly, the consensus achieved might not represent all expert perspectives, and certain guideline details could differ among different participant groups. Fourth, the reporting guideline was developed for scientists, and therefore, only researchers who published VR-related scientific papers were invited. Although several participating researchers also work as clinicians, the lack of variety in stakeholders may have affected the generalizability of the results.

### Future research

Future research could address several topics based on the results of this study. First, studies could increase the scope of this research and, for instance, extend these results to XR or digital health in general, although the current version of the reporting guideline is likely already well suited for describing XR interventions. Also, while our results are limited to therapeutic VR, VR is increasingly being used in diagnostics,^42^ and it might be worthwhile to study this in detail. Second, this study is a small step in advancing research on therapeutic VR, but more steps are needed to mature this relatively new area of research. One of these steps could be the development of a research agenda with the most urgent topics to study in the field of therapeutic VR, to which the current guideline can be applied. The fast-paced evolution of VR warrants continuous updating of this study’s outcomes, including input from lower- and middle-income countries and different VR stakeholders (e.g., clinicians, users, and developers). In addition, new technological advancements, including the use of haptics and eye tracking, are likely to improve and be integrated into therapeutic VR interventions of the future, necessitating further expansion or modification for optimal VR intervention reporting. To ensure continuous relevance, the INVIRTUE group will update the reporting guideline every 5 years, taking into account significant developments within the field.

### Impact

Implementation of this reporting guideline could be achieved in multiple ways. We will notify academic journals and researchers (including Delphi participants) about the completion of the guideline. Furthermore, this reporting guideline is freely available, and we will continue cooperating with EQUATOR to disseminate this reporting guideline. Implementation of this guideline is expected to have an impact on different stakeholders in research on therapeutic VR. Researchers could use the reporting guidelines for transparent and comprehensive intervention reporting. This way, the reporting guideline would support appropriate interpretation, comparison, and pooling of VR interventions. Clinicians could use the reporting guideline to extract essential VR intervention elements (e.g., dosage) required for accurate intervention application, which would ultimately support intervention adoption and effectiveness in clinical practice. Finally, the reporting guideline may also inform other stakeholders, including funding bodies and industry, by improving the clarity and consistency of intervention reporting and facilitating the translation of research findings into real-world applications.

## Conclusion

To conclude, this study reached consensus on key items and categories to report therapeutic VR interventions in scientific papers. These results culminated in the new consensus INVIRTUE VR intervention reporting guideline, which provides the scientific community with a meaningful tool for uniform, detailed, and comprehensive VR intervention description. Utilizing this tool could support researchers in study interpretation, comparison, and replicability, ultimately building a stronger empirical foundation for VR utility and supporting clinical practice in the appropriate application and effective implementation of therapeutic VR in health care.

## Authors’ Contributions

S.S. was the principal investigator of this study, part of the day-to-day committee, and drafted the first version of the article. H.v.G., R.O., B.S., and J.K. were part of the day-to-day committee, provided feedback on the article, and supervised by S.S. M.v.d.H. participated in this project as part of his thesis and was supervised by S.S. and J.K. D.H., L.B., B.D., T.D.G., P.A.v.d.H., M.K., B.S., Z.T., and D.U. were part of the steering committee and provided feedback on the article.

## Data Availability

The data generated during these studies will not be publicly available but will be available upon reasonable request to the corresponding author.

## Abbreviations

eDelphi: Electronic Delphi
EQUATOR: Enhancing the QUAlity and Transparency Of health Research
HMD: Head-mounted display
INVIRTUE: VR intervention reporting international consensus
OSF: Open Science Framework
RATE-XR: Reporting for the early-phase clinical evaluation of applications using extended reality
TIDieR: Template for Intervention Description and Replication
VR: Virtual reality
XR: Extended reality

## Appendix

### Appendix A. Information Letter Delphi Study

#### Development of international reporting guideline for VR intervention studies

##### Purpose.

The purpose of this study is to develop an international reporting guideline for therapeutic head-mounted display (HMD) virtual reality (VR) interventions in health care for participants. With this guideline, we aim to create a standard for reporting VR intervention studies, which will, for example, help in comparing studies.

##### What participation involves.

This study is based on the Delphi methodology. The aim of this method is to reach consensus between experts through three online Delphi rounds. In each of the three rounds, you will be asked to rate proposed items based on a 9-point Likert scale and to classify the individual reporting items in categories. Additionally, in the first round, you will have the opportunity to propose new items for consideration via a free-text box. After the first and second rounds, the answers will be analyzed and you will be provided with a short summary at the start of the second and third rounds, respectively. A participant will only be invited to the next Delphi round when the previous round is completed.

##### Time.

Completing each Delphi round will take approximately 15 min.

##### Consent.

You can consent digitally before filling in the survey.

##### Data usage.

The following personal data will be collected: email address, sex, age, country of residence, VR research context, VR research setting, and experience in VR research. The following research data will be collected: rating of the items and the associated category. Two researchers will code the data independently. The personal data will be saved separately from the research data. Both will be saved in a secure online environment of the HAN University of Applied Sciences. Personal data will be deleted after completion of the first Delphi round. The research data will be saved for 10 years.

##### Confidentiality.

The data will be pseudonymized. One researcher will know which person gave which answer. This procedure will be followed to ensure we know who to contact for the next Delphi rounds. The researchers performing the data analyses will use pseudonymized data. Your answers will be kept confidential, and your personal data will not be shared outside the research team.

##### Compensation.

Participants who finish all three Delphi rounds will be mentioned as authors of the paper as part of the “VR intervention reporting international consensus (INVIRTUE) group.”

##### Withdrawal.

You can withdraw from the study at any moment without any consequences. There is no need for explanation for withdrawal.

##### Contact information.

If you have any questions or remarks about the study, or you wish to get more information, you can contact syl.slatman@han.nl. If you have any questions or complaints regarding privacy, you can contact the HAN University of Applied Sciences’ data protection officer: privacy@han.nl.

### Appendix B. Reporting Items per Delphi Round

**Table:** Round 1

No.	Item	Category	Description	Example
1	Proposed working mechanism	Theory	A description of the proposed working mechanism of the VR intervention	Increasing range of motion or reducing anxiety
2	Target group		A description of whether the VR intervention specifically targets a particular participant group or joint	
3	Intervention time session	Dose	A description of the duration of one session	30 min
4	Intervention duration		A description of the total time period of the intervention	4 weeks
5	Frequency		A description of how many times a day/week/month a session was administered	3 times a week
6	Intensity		A description of the intensity on which the intervention was based	Heart rate, percentage of 1RM
7	Intervention modification		A description of whether the intervention could be modified during the intervention	Alter the intervention scenery during use
8	Hardware	Content	A description of the hardware and its modifications that were used	Oculus Rift 3
9	Application/game		A description of the application/game used for the intervention	Beatsaber or Reality Health
10	Intervention procedure		A description of the explanation of the VR game	
11	Level of immersion		A description of the level of immersion of the VR hardware	Head-mounted display
12	Audio		A description of whether an audio system was used	Headphones
13	Motion tracking		A description of whether the motion of the participants was tracked when using the VR	
14	Embodiment		A description of whether the VR used the sensations of being inside, having, and controlling a virtual body	
15	Feedback mechanism		A description of whether the participant received feedback from the VR and vice versa	Audio-visual feedback
16	Interactivity		A description of whether the VR application was interactive	To which degree the participant can interact with the virtual environment
17	Intervention personalization		A description of whether the participant could, for example, choose which game, scenery, or audio to use	
18	Delivery		A description of where the intervention took place	At home (unsupervised)
19	Body position		A description of whether the intervention was delivered lying, sitting, or standing	
20	Priming and co-interventions		A description of integration of VR and other manipulations	
21	Software for development	Preparation	A description of the software used to make the game/application	Unity3D
22	Safety measures		A description of whether the safety of participants was warranted	Safety harness
23	Intervention instruction		A description of what kind of instruction was given to the participants about the use of the VR	
24	Intervention setup		A description of how the VR hardware and room were set up for the intervention	
25	Costs	Additional	A description of the costs of the VR hardware/software	
26	Side effects/cybersickness		A description of what kind of possible side effects could occur	Dizziness

**Table:** Round 2

No.	Item	Category	Description	Example	Based on item no. previous round/new item
1^a^	Clinical aim	Theory	Description of the clinical aim of the VR intervention	Increasing range of motion or reducing anxiety	1
2^a^	Time per session	Dose	Description of the total duration of one treatment session	30 min	3
3^a^	Intervention duration		Description of the total time period of the intervention	4 weeks	4
4^a^	Frequency		Description of how many times a day/week/month a session was administered	3 times a week	5
5^a^	Intervention setup	Usability	Description of how the VR hardware and room were set up for the intervention and where it took place	At home or in a hospital room	24
6^a^	Application/game	Content	Description or name of the application/game used for the intervention	Beatsaber or Reality Health	9, 10
7^a^	Interactivity		Description whether the VR application was interactive	Whether the participant could interact with virtual avatars	16
8	Proposed working mechanism	Theory	Description of the underpinning neuro/psycho/biological basis for the intervention	How a VR intervention specifically addresses central pain mechanisms for people with chronic pain	1
9	Purpose of VR intervention		Description of the purpose of the VR intervention	Assessment, treatment, or monitoring	New
10	Scientific evidence		Description of the scientific evidence for the proposed working mechanism	Literature supporting these working mechanisms	New
11	Target group	Population	Description whether the VR intervention specifically targets a particular participant group or body region	People with chronic low back pain	2
12	Participant involvement		Description whether the target population was involved in the development of the technology and in which way they contributed	Participants with agoraphobia	New
13	Intervention modification	Content	Description whether the VR intervention has an adaptive level system to increase or decrease the level of the VR intervention and how this system works	Automatic progression in difficulty based on performance of participant	7
14	Intervention personalization		Description whether the participant can adapt the content of the VR intervention	Choice in scenery or audio	17
15	Body position		Description in which body position the VR intervention was applied	Lying, sitting or standing	19
16	Hardware		Description of the hardware	Oculus Quest 3	8
17	Availability/accessibility		Description whether the application/game used in therapy is accessible/available for the public (open source or not)		New
18	Feedback mechanism		Description whether the participant received (audio-visual feedback, tactile, or vestibular) task feedback from the VR		15
19	Motion tracking		Description whether the motion of the participants was tracked when using the VR		13
20	Embodiment		Description whether the participant embodied an avatar		14
21	Co-interventions		Description of other interventions provided during the VR intervention period	Homework exercises	20
22	VR time		Description of the time that the VR intervention was used during the treatment	15 min	3
23	Audio		Description whether an audio system was used	Headphones	12
24	Presence		Description of the degree to which the participant had the feeling of being in the virtual environment		11
25	Intervention instruction	Usability	Description of what kind of instruction was given to the participants about the use of the VR and the time needed for the instruction	10-min, oral instruction	23
26	Technical requirements		Description of required materials and services to use the VR intervention	Use of Wi-Fi, laptop	8
27	Provision/delivery		Description of who provided/delivered the VR intervention	(Health care) professional, or that the VR intervention was self-administered	18
28	Preparation time		Description of how much time the setup of the system took before the VR intervention could start	15 min	23
29	Professional training		Description of the training given for those delivering the intervention	1-h, online training	New
30	Costs		Description of the costs of the VR hardware, software, and/or additional costs such as cleaning supplies		25
31	Safety measures	Safety	Description of whether the safety of participants was warranted	Safety harness, supervision	22
32	Possible adverse events		Description of issues that may be encountered during the intervention	Side effects, cybersickness, falling	26
33	Data security		Description of how and where the data about the participant is collected and processed	In local, secured storage	New

Excluded from round 1: intensity (item 6) and software for development (item 21).

a Included in the previous round and tested for stability.

**Table:** Round 3

No.	Item	Category	Description	Example	Based on item no. previous round
1^a^	Clinical aim	Theory	Description of the clinical aim of the VR intervention	Increasing range of motion or reducing anxiety	1, 9
2	Proposed working mechanism		Description of the underpinning neuro/psycho/biological working mechanism for the intervention	How a VR intervention specifically addresses central pain mechanisms for participants with chronic pain, and whether this working mechanism is supported by evidence	8, 10
3^a^	Application/game	Content	Description, illustration, and name of the application/game used for the intervention	Beatsaber or Reality Health	6
4^a^	Interactivity and feedback		Description of whether and how the VR application was interactive and provided (audio-visual feedback, tactile, or vestibular) task feedback	Whether and how the participant could interact with virtual avatars	7, 18
5	Modification, personalization, and tailoring		Description of whether and how the VR intervention had the option to modify the intervention for the specific participant	Automatic progression in difficulty based on the performance of the participant	13, 14
6	Availability/accessibility		Description of whether the application/game used in therapy is accessible/available for the public (open source or not)	Off-the-shelf application	17
7	Hardware		Description of the used hardware and services (e.g., Wi-Fi), including HMD, audio, and motion tracking equipment	Oculus Quest 3	16, 19, 23, 26
8	Co-interventions		Description of other interventions provided during the VR intervention period	Homework exercises	21
9^a^	Dosage	Deployment	Description of the intervention duration, frequency, and time per session	4 week intervention, three times per week for 15 min	2, 3, 4, 22
10^a^	Setting and supervision		Description of where and how the intervention took place and whether this was supervised (including safety considerations)	At home or in a hospital room supervised by a physiotherapist	5, 15, 31, 33
11	Instruction		Description of what kind of instruction was given to the participant about the use of the VR, by whom and the time needed for the instruction	10-min, oral instruction by a nurse	25, 27
12	Preparation time		Description of how much time the setup of the system took before the VR intervention could start	15 min	28
13	Professional training		Description of the training given to the professional delivering the intervention	1-h, online training	29
14	Target group	Population	Description of whether the VR intervention specifically targets a particular participant group or body region	Participants with chronic low back pain	11
15	Participant involvement		Description of whether the target population was involved in the development of the intervention and in which way they contributed	Participants with agoraphobia contributed to the intervention content description	12
16	Possible adverse events	Safety	Description of issues that may be encountered during the intervention	Side effects like cybersickness, falling	32

Excluded from round 2: costs (item 30), presence (item 24), and embodiment (item 20).

a Included in the previous round and tested for stability.

### Appendix C. Demographic Information Nonresponders

**Table:**

	Expert panel (*n* = 61)	Nonresponders (*n* = 43)	Total responders (*n* = 104)
Sex, female, *n* (%)	32 (52)	23 (53)	55 (53)
Age, mean (SD)	38.6 (9.7)	40.5 (10.1)	39.4 (9.8)
Country of origin, *n* (%)			
The Netherlands	26 (43)	13 (30)	39 (38)
The United Kingdom	7 (11)	1 (2)	8 (8)
The United States	5 (8)	6 (14)	11 (11)
Germany	4 (7)	0 (0)	4 (4)
Belgium	4 (7)	1 (2)	5 (5)
Australia	3 (5)	1 (2)	4 (4)
Switzerland	3 (5)	2 (5)	5 (5)
Denmark	2 (3)	0 (0)	2 (2)
Ireland	2 (3)	0 (0)	2 (2)
Italy	2 (3)	6 (14)	8 (8)
Canada	1 (2)	3 (7)	4 (4)
Chile	1 (2)	0 (0)	1 (1)
Poland	1 (2)	0 (0)	1 (1)
France	0 (0)	3 (7)	3 (3)
Spain	0 (0)	2 (5)	2 (2)
Czech Republic	0 (0)	2 (5)	2 (2)
Finland	0 (0)	1 (2)	1 (1)
Hungary	0 (0)	1 (2)	1 (1)
Norway	0 (0)	1 (2)	1 (1)
VR research discipline, *n* (%)^a^			
Pain management	24 (39)	15 (35)	39 (38)
Physical rehabilitation	20 (33)	20 (47)	40 (38)
Mental health therapy	15 (25)	17 (40)	32 (31)
Other	24 (39)	14 (33)	38 (37)
VR research setting, *n* (%)^a^			
Out-hospital	42 (69)	23 (53)	65 (63)
In-hospital	32 (52)	25 (58)	57 (55)
Fundamental	19 (31)	17 (40)	36 (35)
Years’ VR experience, mean (SD)	6.0 (4.3)	7.5 (5.2)	6.6 (5.0)

a Multiple options are possible.

SD, standard deviation.

### Appendix D. Customizable Version of INVIRTUE Reporting Guideline

**Table:**

	Item	Description	Example
Theory	1. Clinical aim	Description of the clinical aim of the VR intervention	The aim of the VR intervention was to increase the range of motion of the user’s shoulder
	2. Proposed working mechanism	Description of the proposed underpinning neuro/psycho/biological working mechanism of the VR intervention	The proposed working mechanism of the VR intervention was distraction
	3. Proposed user group	Description of whether the VR intervention was developed for a particular user group or body region	The provided VR intervention was originally developed for users with chronic low back pain
Content	4. Application/game	Description, illustration, and name of the VR intervention	The VR intervention “Beatsaber” was used (see Fig. 1). In this intervention, users use VR controllers to slice through flying colorful blocks in sync with music
	5. Interactivity and feedback	Description of whether and how the VR intervention was interactive and provided (audio-visual, tactile, or vestibular) task feedback	The VR intervention included interaction with avatars and provided audio-visual feedback
	6. Tailoring	Description of whether and how the VR intervention had the option to be tailored (e.g., using different levels or personalization) to the specific user	VR intervention tailoring was possible through automatic progression in difficulty based on the performance of the user
	7. Availability	Description of whether the VR intervention is available to the public (open source or not)	The VR intervention used was an available off-the-shelf intervention
Deployment	8. Hardware	Description of the hardware and services (e.g., Wi-Fi) used, including HMD, audio, and motion tracking equipment	The VR intervention was provided using the standalone Oculus Quest 3 HMD VR headset with built-in spatial audio
	9. Dosage	Description of the VR intervention duration, frequency, and time per session	The VR intervention had a duration of 4 weeks, three times per week for 15 min per session
	10. Setting and supervision	Description of the location where the VR intervention took place and whether this was supervised	The VR intervention was provided in a hospital room supervised by a physiotherapist
	11. Instruction	Description of what kind of instruction was given to the user about the use of the VR intervention, by whom, and the time needed for the instruction	Users who received the VR intervention were given a 10-min verbal instruction by a nurse
	12. Preparation time	Description of how much time the setup of the system took before the VR intervention could start	Setup of the VR intervention took approximately 15 min
	13. Professional training	Description of the training given to the professional providing the VR intervention	Physiotherapists who provided the VR intervention received a 1-h online training
Development	14. End-user involvement	Description of whether the end-user (e.g., user or provider) was involved in the development of the VR intervention and in which way they contributed	Users with agoraphobia contributed to the intervention content description
Safety	15. Anticipated adverse events	Description of issues that may be encountered during the VR intervention and utilized safety considerations	To diminish the risk of falling during the VR intervention, a safety harness was used
Context	16. Co-interventions	Description of other interventions provided during the VR intervention period	The users who received the VR intervention simultaneously received weekly psychotherapy
